# 2136. In Vitro Activity of Nacubactam (OP0595) Combined with Cefepime against Carbapenem-resistant Enterobacterales Isolated in Japan

**DOI:** 10.1093/ofid/ofad500.1759

**Published:** 2023-11-27

**Authors:** Katsunori Yanagihara, Hiroshige Mikamo, Kazuhiro Tateda, Yohei Doi, Hiroki Ohge, Satoshi Takahashi, Kenichiro Kondo

**Affiliations:** Nagasaki University, Nagasaki, Nagasaki, Japan; Aichi Medical University, Aichi, Aichi, Japan; Toho University, Tokyo, Not Applicable, Japan; Fujita Health University School of Medicine, Toyoake, Aichi, Japan; Hiroshima University Hospital, Hiroshima, Hiroshima, Japan; Department of Infection Control and Laboratory Medicine, Sapporo Medical University School of Medicine, Sapporo, Hokkaido, Japan; Meiji Seika Pharma Co., Ltd., Tokyo, Tokyo, Japan

## Abstract

**Background:**

Nacubactam is a new non-β-lactam diazabicyclooctane β-lactamase inhibitor that inhibits penicillin binding protein 2 (PBP2) of Enterobacterales and acts synergistically as a β-lactam enhancer when combined with β-lactams. Nacubactam is under development for the treatment of serious Gram-negative infections. We evaluated the in vitro activity of cefepime-nacubactam against carbapenem-resistant Enterobacterales (CRE) clinical strains isolated at six medical facilities in Japan.

**Methods:**

Activity of cefepime-nacubactam (1:1 ratio) was tested against 376 CRE (MIC of meropenem or imipenem: ≥2 mg/L). The MICs were evaluated by the broth microdilution method according to the CLSI using frozen-panels (Eiken Chemical Ltd., Japan) containing various antimicrobial agents. The percentage susceptible (%S) for cefepime-nacubactam was calculated using the CLSI susceptible breakpoint of cefepime.

**Results:**

The collection included 184 carbapenemase-producing Enterobacterales (CPE) strains and 192 carbapenemase-non-producing Enterobacterales (non-CPE) strains. Against CPE (*Escherichia coli*, 27; *Klebsiella* spp., 89; *Enterobacter* spp.; 56; others, 12), the MIC_50_/MIC_90_ and %S were 8/32 mg/L and 4.9%S for meropenem, 4/32 mg/L and 29.3%S for imipenem, 32/ >128 mg/L and 31.1%S as 8 mg/L for cefepime and 2/4 mg/L and 95.7%S for cefepime-nacubactam, respectively. Against non-CPE (*E. coli*, 16; *Klebsiella* spp., 80; *Enterobacter* spp., 76; others, 20), the MIC_50_/MIC_90_ and %S were 0.5/8 mg/L and 53.6%S for meropenem, 2/8 mg/L and 12.0%S for imipenem, 2/ >128 mg/L and 66.7%S as 8 mg/L for cefepime and 0.25/4 mg/L and 99.0%S for cefepime-nacubactam, respectively. The MIC_50_/MIC_90_ and %S of cefepime-nacubactam was 2/4 mg/L and 94.9%S against 156 MBL-producing CRE, and 0.5/2 mg/L and 100%S against non-MBL-producing CRE including 14 KPC and 8 OXA-producing strains and 6 strains producing other carbapenemases. Nacubactam alone showed limited activity against CPE (MIC_50_/MIC_90_, 4/ >16 mg/L) and non-CPE (MIC_50_/MIC_90_, >16/ >16 mg/L).

In vitro activity of cefepime-nacubactam against CRE isolated in Japan

**Figure:**
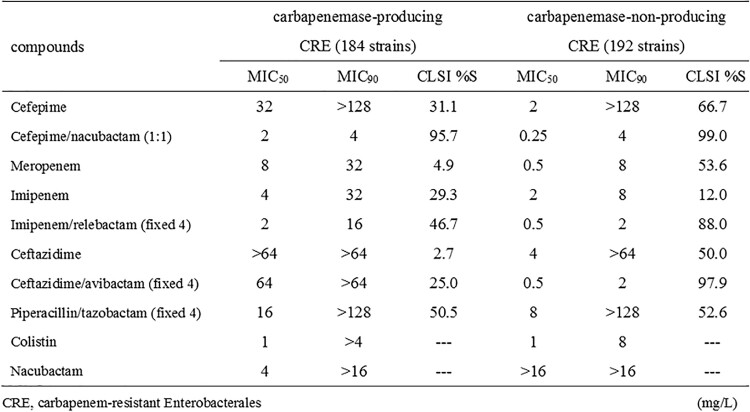

**Conclusion:**

Cefepime-nacubactam demonstrated potent activity against both CPE and non-CPE/CRE clinical strains. This is the first study to report the activity of cefepime-nacubactam against carbapenemase-non-producing CRE, which accounts for 80% of CRE in Japan.

**Disclosures:**

**Katsunori Yanagihara, MD, PhD**, FUJIFILM Toyama Chemical Co., Ltd.: Commissioned research|KYORIN Pharmaceutical Co.,Ltd.: Commissioned research **Hiroshige Mikamo, M.D, Ph.D**, Asahi Kasei Pharma Corporation: Grant/Research Support|Merck Sharp & Dohme: Honoraria|Pfizer Inc.: Grant/Research Support|Pfizer R&D Japan: Honoraria|Sumitomo Pharma Co., Ltd.: Grant/Research Support|Sumitomo Pharma Co., Ltd.: Honoraria **Yohei Doi, MD, PhD**, bioMerieux: Advisor/Consultant|FujiFilm: Advisor/Consultant|Gilead: Advisor/Consultant|Gilead: Honoraria|GSK: Advisor/Consultant|Meiji Seika Pharma: Advisor/Consultant|Moderna: Advisor/Consultant|Moderna: Honoraria|MSD: Advisor/Consultant|MSD: Honoraria|Shionogi: Advisor/Consultant|Shionogi: Grant/Research Support|Shionogi: Honoraria

